# Infantile hemangioma models: is the needle in a haystack?

**DOI:** 10.1186/s12967-023-04144-0

**Published:** 2023-05-06

**Authors:** Meng Kong, Yanan Li, Kai Wang, Shisong Zhang, Yi Ji

**Affiliations:** 1 Division of Oncology, Department of Pediatric Surgery, West China Hospital, Sichuan University, #37# Guo-Xue-Xiang, Chengdu, 610041 China; 2 Department of Pediatric Surgery, Children’s Hospital Affiliated to Shandong University, #23976# Jingshi Road, Jinan, 250022 China

**Keywords:** Infantile hemangioma, Pathogenesis, 3D microtumor, Transplantation model

## Abstract

Infantile hemangioma (IH) is the most prevalent benign vascular tumor in infants, with distinct disease stages and durations. Despite the fact that the majority of IHs can regress spontaneously, a small percentage can cause disfigurement or even be fatal. The mechanisms underlying the development of IH have not been fully elucidated. Establishing stable and reliable IH models provides a standardized experimental platform for elucidating its pathogenesis, thereby facilitating the development of new drugs and the identification of effective treatments. Common IH models include the cell suspension implantation model, the viral gene transfer model, the tissue block transplantation model, and the most recent three-dimensional (3D) microtumor model. This article summarizes the research progress and clinical utility of various IH models, as well as the benefits and drawbacks of each. Researchers should select distinct IH models based on their individual research objectives to achieve their anticipated experimental objectives, thereby increasing the clinical relevance of their findings.

## Introduction

Infantile hemangioma (IH) is the most common benign vascular tumor in infants, with an incidence rate of approximately 4–5% [1]. Infants born prematurely and with low birth weight have a higher incidence rate, and the male to female ratio is approximately 1:3 [2]. The clinical presentation of IH varies depending on its location, depth, and stage of advancement. IH can occur anywhere in the body, including the internal organs, but it is more common in the head and neck, trunk, and limbs [3]. Although 90% of IHs resolve on their own, larger and faster-growing IHs may leave permanent pigmentation, vascular dilatation, fibrofatty tissue buildup, and scarring following regression. IH in specific sites can lead to major problems such as organ failure, visual impairment, restricted joint movement, breathing difficulties, and even death [4–6]. Furthermore, IH might have a negative impact on the quality of life and psychological health of the affected child and their family [7, 8]. IH is distinguished by a distinct growth pattern and is classified into three stages: the proliferative stage, characterized by a significant increase in endothelial cell proliferation; the stable stage, characterized by a gradual decrease in endothelial cell proliferation; and the involution stage, during which the tumor regresses and the vascular tissue is replaced by fibrofatty tissue [1]. Medication, surgery, and laser therapy are currently the most common clinical treatment options for IH [9]. Propranolol, a nonselective beta-adrenergic receptor blocker, was accidentally identified in 2008 by Léauté-Labrèze et al. to effectively reduce and/or limit the growth of IH [10]. Propranolol has now become the first choice for treating problematic IH requiring systematic therapy [11]. Propranolol has been discovered to have many modes of action for IH, including increasing vasoconstriction, decreasing cell development, and triggering cell death [12]. Although propranolol’s therapeutic efficacy for IH has been extensively recognized internationally, its negative effects limit its wider use. Symptoms such as bradycardia, wheezing, sleepiness, diarrhea, hypoglycemia, and hypotension have been reported in studies [13, 14]. In addition, over 10% of patients acquire resistance to propranolol treatment, and 19% experience recurrence [15, 16]. Thus, new therapeutic targets for IH and new drugs with substantial clinical value are needed.

The pathophysiology of IH is unknown. It has been reported that the occurrence of IH may be caused by the mutation of key genes of somatic cells leading to the clonal growth of stem cells [17, 18]. Other studies have found that tissue hypoxia and the renin-angiotensin system (RAS) may also be independent and important risk factors for IH [19–21]. In addition, epidemiological factors such as preterm birth, low birth weight, placental dysfunction and preeclampsia are also closely associated with the occurrence of IH [22, 23]. Although there may be one or more causal reasons for IH, excessive neovascularization is a common trait in IH pathogenesis.

As research on the pathophysiology of IH advances, however, there are still more intricate networks to be investigated. The development of IH models is a critical tool and strategy for researching IH pathophysiology and therapy options. The lack of model systems and research approaches that successfully imitate human disease is now the key obstacle in IH research. With the ongoing improvement of experimental methodologies in recent years, several types of IH models, such as cell suspension inoculation models, viral gene transfer models, tissue block transplantation models, and in vitro culture tumor cell sphere models, have arisen. We discussed the application breadth and advantages and disadvantages of each model, providing essential reference values for the selection of acceptable IH models for future study to further unravel the etiology and pathogenesis of IH.

## Model of cell suspension implantation

**Fig. 1:**
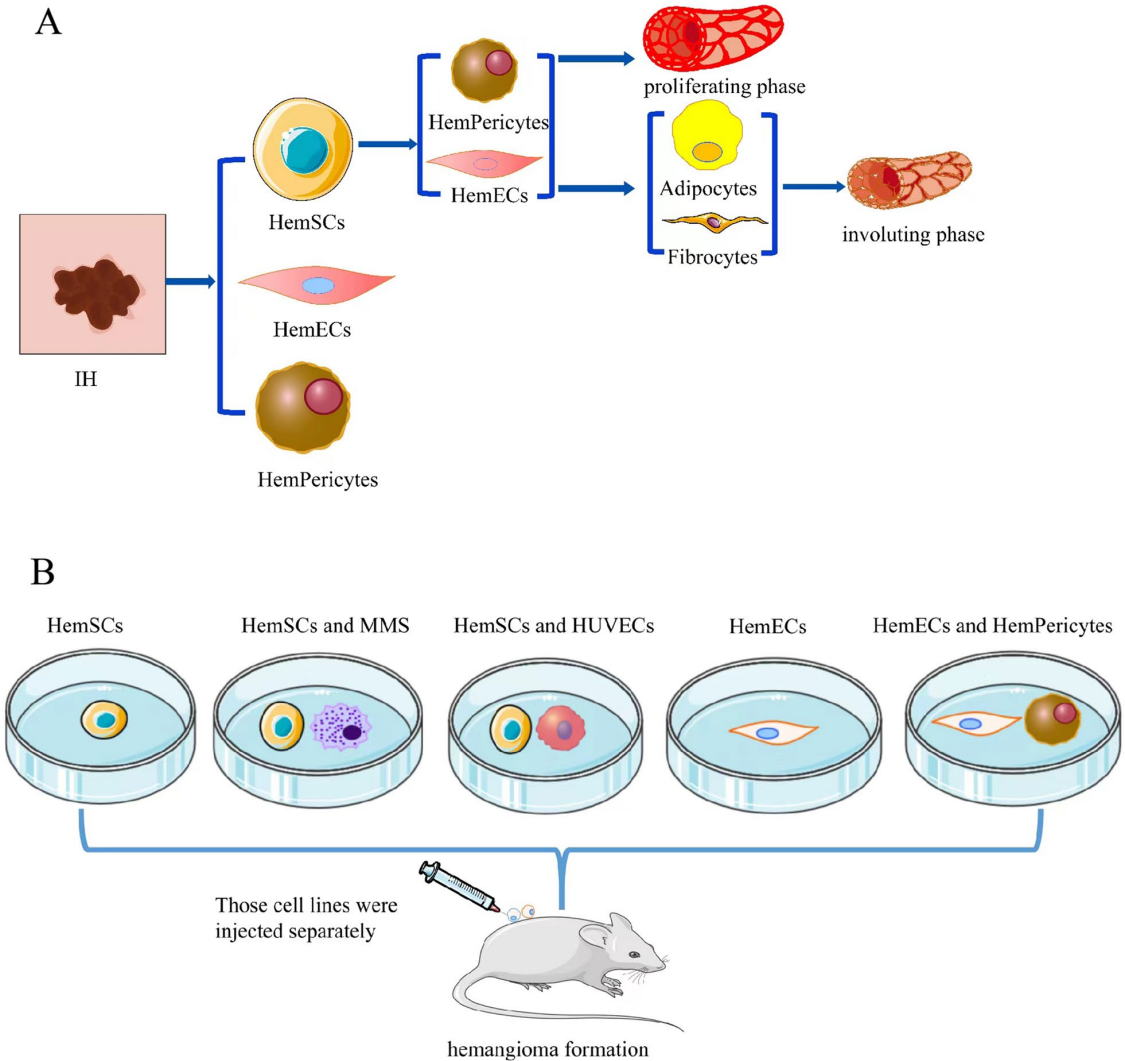
**A** The primary components of IH. The major components of IH include HemSCs, HemECs and Hempericytes. In the proliferative stage of IH, HemSCs are mainly differentiated into HemECs and Hempericytes to promote angiogenesis. In the phase of IH regression, adipocytes and fibrocytes were mainly differentiated. **B** Model of cell suspension implantation. Cell suspension injection has become the most commonly used method for IH model construction. HemSCs, HemSCs and MMS, HemSCs and HUVECs, HemECs, HemECs and Hempericytes were mixed with matrix gel with syringe implantation to prepare cell suspensions. Then, they were injected subcutaneously into the back of nude mice. The implanted cells proliferated and differentiated to form local hemangiomatous masses

Angiogenesis and vasculogenesis have been shown to be essential pathways in the development of IH [24]. Angiogenesis is defined as the development of new blood vessels from preexisting vessels, which requires basement membrane disintegration, endothelial cell migration, tube formation, and recruitment of perivascular cells. Vasculogenesis, on the other hand, is the process by which blood vessels are formed from stem or progenitor cells [25]. Hemangioma stem cells (HemSCs), hemangioma progenitor cells, hemangioma endothelial cells (HemECs), and hemangioma-derived pericytes (HemPericytes) are the primary components of IH (Fig. 1A). HemSCs primarily differentiate into HemECs and HemPericytes during the proliferation stage of IH to promote blood vessel creation, while they mostly differentiate into fibrofatty cells during the involution stage [18]. As a result, the cell suspension injection method, in which cells (HemSCs, HemECs) cultured in vitro are suspended at a specific concentration in a matrix gel to prepare a cell suspension, which is then injected subcutaneously into nude mice, has become the most commonly used IH model construction method. The transplanted cells have the ability to multiply and differentiate locally, resulting in IH-like tumors (Fig. 1B).

### HemSC model

According to research on the formation of hemangioma endothelial cells, they may be derived from defective and immature stem cells [26]. Khan et al. [27] reported for the first time in 2008 the isolation of multipotent HemSCs from proliferative IH tissue for the creation of an IH animal model. The researchers implanted CD133+ immunomagnetic bead-selected HemSCs under the skin of naked mice for 7 days, and new blood vessel creation was observed in the implanted location. Vascular tissue blocks expressing the IH-specific immunomarker glucose transporter-1 (GLUT-1) could be produced again in subsequent recipients. The number of blood vessels dropped and deteriorated into fat tissue 2 months after implantation. As a result, their study initially identified CD133+ HemSCs as the source of IH. When CD133+ HemSCs were implanted subcutaneously into immunodeficient mice, GLUT-1-positive blood vessels developed within 1–7 days, which are particular indicators of IH. Itinteang et al. [28] discovered primitive mesoderm cells in IH using HemSCs labeled with green fluorescence protein (GFP) in naked mice. The identification of HemSCs allows us to investigate new pathogenic mechanisms of IH at the molecular and cellular levels, laying the groundwork for future research into the biological properties of HemECs and the development of new therapeutic approaches [29]. This model’s development can replicate the evolution of IH and examine the processes of its incidence, growth, and regression. The downside is that in practice, there are significant equipment requirements for selecting HemSCs, high antibody pricing, and challenges managing sorting rates. Additionally, the model did not show rapid expansion of the IH. This could be because critical cell components have been lost. Of course, the particular reasons and regulatory mechanisms need to be investigated further.

Xu et al. [30] discovered that spalt-like transcription factor 4 (SALL4)+ and CD133+ cells were substantially more abundant in proliferative vascular tumor specimens than in degenerated tumors after isolating, identifying, and culturing IH stem cells and conducting in vitro and in vivo tests with animal models. Tumor sphere creation techniques were utilized to develop vascular tumor cells in vitro. Cells in IH tumor spheres displayed several stem/progenitor cell markers, including SALL4, kinase insert domain receptor (KDR), and CD133, as well as high amounts of GLUT-1 and vascular endothelial growth factor (VEGF). These cells may self-renew and develop into endothelial cells, both of which are characteristics of tumor stem cells. Subcutaneous injection of IH tumor sphere cells into immunocompromised NOD-SCID mice resulted in GLUT-1-positive and CD31-positive tumors with the same cell proliferation, differentiation, and degeneration processes seen in human IH. They discovered that a large number of HemSCs can be cloned in vitro, and the establishment of the mouse model opens up new opportunities for the development of new therapeutic medications. Lv et al. [31] expanded on this method by using proliferative IH specimens and developing a more comprehensive and fast HemSC identification method based on collagenase digestion and CD133 immunomagnetic bead sorting. In vitro, these cells can create a mesh-like structure similar to the arterial wall and can be coaxed to develop into bone and fat cells. They induce lesions similar to IH when injected subcutaneously in naked mice. This technique can generate a high number of IH stem cells in vitro, setting the groundwork for further research into the properties of IH stem cells and their broader applications. The downside is that this model did not reflect the rapid growth seen in infantile hemangioma during the IH creation phase, and more work is needed to improve it. Because HemSCs have significant proliferation and differentiation capacities, they can differentiate into HemECs and HemPericytes, which are key components of the proliferative phase of IH when injected subcutaneously into nude mice. Previous research has revealed that mesenchymal support cells are required for normal human endothelial cell vascular network development [32]. Boscolo et al. [33] discovered that when HemSCs are transplanted alone under the skin of nude mice without mesenchymal support cells, they can generate blood vessels in vivo. They believe that more research is needed to establish whether the genetic mechanisms that contribute to vascular formation in HemSCs vary when the external environment changes.

**Fig. 2:**
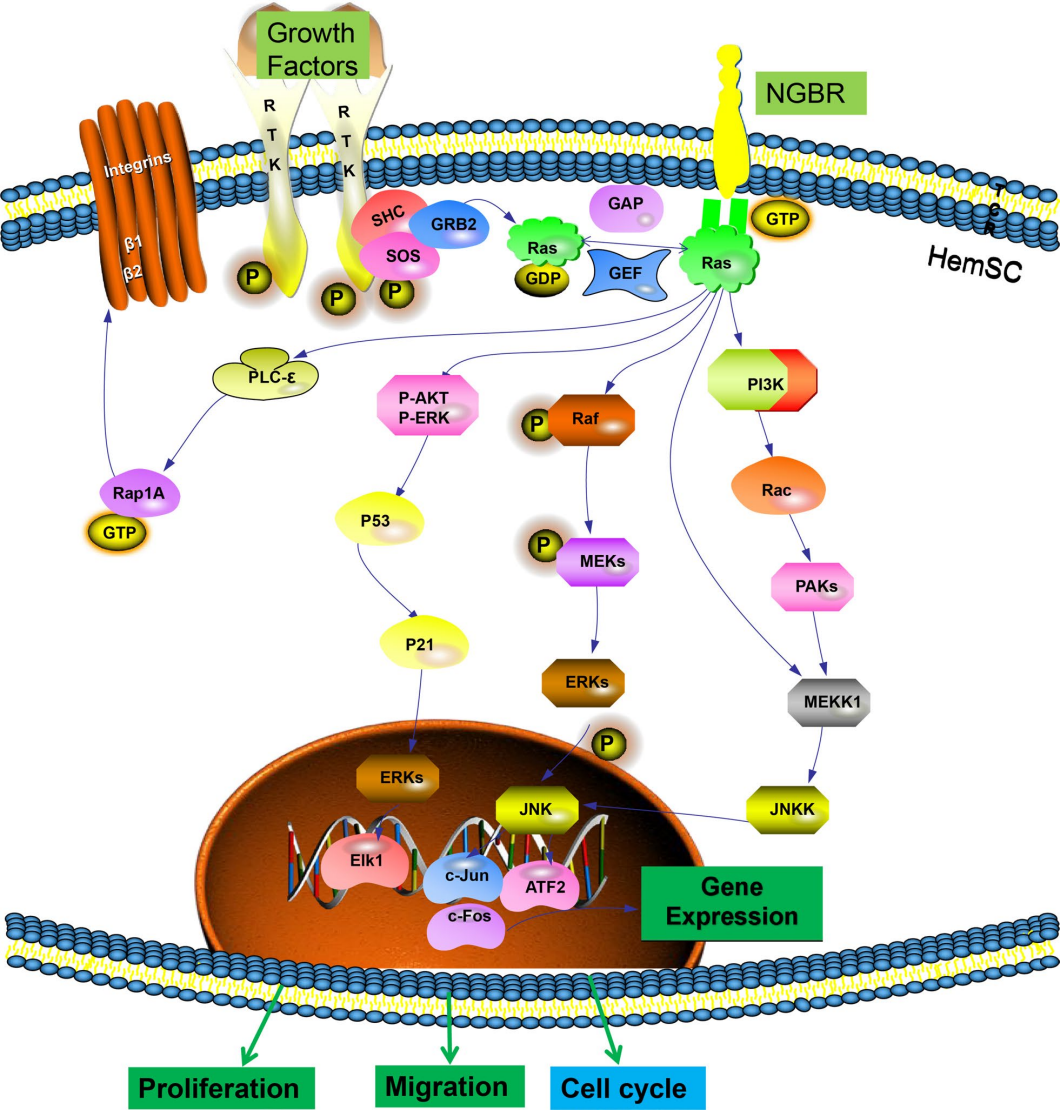
The NGBR/Ras signaling pathway promotes angiogenesis. NGBR is a transmembrane protein. NGBR promotes the migration and proliferation of HemSCs by activating the Ras signaling pathway. NGBR acts as a Ras regulator in controlling the growth and differentiation of HemSCs. NGBR activates multiple downstream signaling pathways to promote angiogenesis and migration by activating Ras in HemSCs. NGBR also promotes the cell cycle and ultimately angiogenesis. The three important signaling pathways are as follows: (1) After activating Ras, NGBR further activates the Raf/ERK/MEK/ERK signaling pathway, promotes nuclear gene expression, promotes cell proliferation and migration, and promotes G1 to S phase transition in the cell cycle. (2) NGBR activates the Ras/PI3K/PAK/MEKK1 signaling pathway to promote cell proliferation and migration. (3) NGBR can also activate the AKT/ERK/P53/p21 signaling pathway, promote cell proliferation and migration, and thus promote angiogenesis. In addition, several important growth factors (VEGF, PDGF, FGF2, and EGF) promote cell proliferation and migration by binding to their respective RTK receptors to activate protein tyrosine kinase activity within the receptors, which then activates Ras and Ras-dependent signal transduction cascades in the cell membrane. NGBR regulates the Ras signaling pathway, while PTK growth factors activate HemSC proliferation and migration. Ras, as a core gene in the NGBR pathway, plays an important role in promoting vascular growth. Phosphatidylinositol-3 kinase (PI3K), p21-activated kinase (PAK), mitogen-activated kinase kinase 1(MEKK1), platelet-derived growth factor (PDGF), fibroblast growth factor 2 (FGF2), epidermal growth factor (EGF).

According to the most recent studies, the nonadrenergic stereoisomers of propranolol and atenolol have inhibitory effects on the vascularization of IH [34]. Caroline et al. [35] discovered that R(−) propranolol and R(−) atenolol hindered HemSC differentiation into HemECs and vascularization in a mouse IH model. These findings provide valuable recommendations for optimizing the use of a nonselective β-adrenergic receptor (β-AR) antagonist in the treatment of IH while minimizing side effects. NOGOB is a member of the omental protein family, and its extracellular domain can act as an inducer of endothelial cell activation [36]. NOGOB receptor (NGBR) is considered to be a specific receptor for NOGOB to stimulate endothelial cell migration and angiogenesis [37]. Hu et al. [38] discovered that the NOGOB receptor NGBR is strongly expressed during the proliferative phase of IH but not during the degenerative phase, implying that NGBR may play a role in regulating the formation of vascular tumors. Furthermore, they investigated the effects of NGBR knockdown on the biological activity of HemSCs and discovered that NGBR knockdown can suppress cell proliferation, migration, and invasion, as well as lower the activation of the ras protein and receptor tyrosine kinase (RTK)-mediated signaling pathways (Fig. 2). Nevertheless, neither vascularization nor adipocytes were found in the NGBR-knockdown transplanted tissue, demonstrating that NGBR is required for HemSCs to develop into blood vessels and adipocytes in vivo and plays a critical role in controlling HemSC proliferation and differentiation. Munabi et al. [39] discovered that HemSCs in proliferative IH express both β_1_-AR and β_2_-AR, as well as the involvement of β-ARs and downstream pathways in mediating propranolol effects. Propranolol administration lowered cyclic AMP (cAMP) levels in isolated HemSCs in a dose-dependent manner and activated the mitogen-activated protein kinase (MAPK) pathway downstream of β-ARs. Propranolol was reported to minimize aberrant vascular dilatation and boost p-extracellular signal regulated kinase (ERK) expression after transplanting HemSCs into a nude mouse subcutaneous model. In conclusion, the effect of propranolol on HemSCs is partially mediated by β_2_-AR inhibition, and it has been shown that the β_2_-AR signaling pathway, as well as downstream cAMP and MAPK pathways, plays crucial roles in HemSC pathophysiology. With these investigations, it is evident that this model is widely employed in fundamental experiments, providing crucial options for explaining the mechanism by which propranolol treats IH and identifying new targets.

### HemSCs combined with monocyte-macrophage cell (MMS) line model

After phenotypic polarization, macrophages play specific roles in the immune system, as demonstrated by scientific research. Macrophages can be polarized into classically activated macrophages (M1 polarization) or selectively activated macrophages (M2 polarization) based on microenvironmental stimuli [40]. According to previous studies, macrophages are the primary cellular component in the pathogenesis of IH, with the majority of macrophages located in the stroma of IH. Wang et al. [41] discovered that the expression of M2-polarized macrophages was much higher during the proliferation phase of inflammatory hepatitis than during the regression phase, as evidenced by the high density of CD68/CD163 cells during the proliferation phase. Thus, it is hypothesized that M2-polarized macrophages may play a unique role in the pathogenesis of IH. Research indicates that macrophages produced from monocytes can boost the survival of many stem cell types and regulate their development and differentiation capacities [42]. In their study of the effects of macrophages on HemSCs in vivo and in vitro, Zhang et al. [43] discovered that M1- and M2-polarized macrophages stimulate the proliferation of HemSCs and inhibit adipogenesis by activating the protein kinase B/(PKB/Akt) and extracellular signal-regulated kinase 1/2 (ERK1/2) signaling pathways. Furthermore, endothelial development of HemSCs is promoted by M2-polarized macrophages. To create an animal model, they also combined human monocytes and HemSCs and injected them subcutaneously into nude mice, which boosted microvascular density and slowed the production of adipose tissue. These results demonstrate that macrophages in IH support the growth of tumors by promoting stem cell proliferation and angiogenesis. Targeting macrophages may be a promising method for accelerating the regression of IH. The advantages of constructing a nude mouse model with HemSCs and macrophages as opposed to simply injecting HemSCs are evident based on the preceding research. These advantages include a high degree of similarity to human IH models, higher cell proliferation activity and expression levels of cell density, microvascular density, and CD31 in tissue during the early stages of IH. The regression procedure in the later phases of IH is quite sluggish, and this strategy can substantially overcome the limitations of building a model with HemSCs alone.

### HemSCs combined with human umbilical vein endothelial cells (HUVECs) model

HUVECs have been shown in studies to have the potential for stem cells and certain vascular formation abilities, are regarded as a significant reference indicator for endothelial cell vascular formation function and are frequently utilized in endothelial cell research [44, 45]. Mai et al. [46] discovered that when both HemSCs and HUVECs were injected subcutaneously into nude mice, the vascularization ability of HemSCs was greatly improved, with a considerable rise in GLUT-1-positive neovascularization. In nude mice, however, essentially no blood vessels developed when HUVECs were injected alone. HemSCs and HUVECs work together to promote vascular development. Their findings imply that HemSCs are primitive mesoderm-derived stem cells with significant vascular formation potential that can be fostered in nude mice by coinjection with HUVECs to facilitate IH. Zhang et al. [47] combined HemSCs and HUVECs and injected them subcutaneously into nude mice, followed by varying dosages of estrogen. The combination injection of estrogen considerably increased endothelial cell production, differentiation, and proliferation, lengthened tumor formation time and was more similar to the process of IH proliferation, according to the study. We can conclude from the aforementioned results that coculture of HemECs and HUVECs, along with estrogen injection, can greatly increase the development of IH in animal models, which has a high similarity to human IH and builds a sound experimental foundation for future IH research. The downside of this model is that the number of neovascularizations did not reach the optimal level, the vascular density did not reach the density of human IHs, and the accuracy still needs to be improved.

### HemEC model

Normally, vascular endothelial cells remain dormant, but they can proliferate locally in response to injury or pathogenic stimuli. Anti-angiogenic factors can prevent angiogenesis and restore the natural quiescent state, which is a highly complex process regulated by pro- and anti-angiogenic factors [48]. Variations in these variables caused by environmental factors or alterations can impact angiogenesis. IH is caused by the uncontrolled growth of vascular endothelial cells, which serve as the disease’s signature cells [49]. In the process of IH formation, stimuli arising from alterations or improper expression of specific genes that control vascular endothelial cells may result in aberrant angiogenesis, while the ability of vascular endothelial cells to undergo apoptosis is diminished [50]. Pan et al. [51] implanted HemECs into the subcutaneous tissue of nude mice, and the rate of tumor growth was 100%. The tumor grew swiftly, had a smooth surface, distinct borders, and noninvasive growth and was morphologically, physiologically, and histopathologically comparable to human IH.

One of the pyruvate kinase isoenzymes, pyruvate kinase M2 isoform (PKM2), is an essential glycolytic enzyme in cancer cells [52]. PKM2 has been found to have an impact on cancer metastasis, invasion, the cell cycle, and cell proliferation in earlier research [53]. PKM2 can increase angiogenesis to support tumor growth and progression, according to a recent study [54]. Yang et al. [55] mixed HemECs with overexpressed or knocked-down PKM2 with HUVECs and injected the mixture into the subcutaneous tissue of the left upper limb of 4-week-old male nude mice. Compared to the control group, knocking down PKM2 had the opposite effect on the color of the hemangioma transplants and the increase in VEGF compared to overexpression of PKM2. It can inhibit the growth of hemangioma. These results show that PKM2 is an important part of how hemangioma worsens. If this model is proven to work, it will be an important choice for the process of pathological evolution, pathogenesis, and the creation of new drugs for IH.

Pluripotent stem cells, which are differentiated cells with stem cell-like properties, can adopt stem or progenitor cell-like phenotypes to adapt to microenvironmental changes, such as stress or injury [56]. They are especially prevalent in the liver and pancreas of adults. After specific toxin injury, pluripotent stem cells in the adult liver can produce biliary epithelial cells and hepatocytes, effectively promoting liver regeneration [57]. After pancreatic duct ligation, acinar cells in the adult pancreas can exhibit multipotent progenitor cell characteristics and produce ducts and endocrine cells [58]. These new findings demonstrate the significance of pluripotent stem cells in tissue and organ regeneration and repair. Huang et al. [59] isolated HemECs and HemSCs from IH patient specimens to determine the underlying causes of hemangioma cell development. In vitro, HemECs could be induced to differentiate into endothelial cells, HemPericytes, smooth muscle cells, and adipocytes, displaying stem cell characteristics. Finally, these differentiated cells were combined with matrix gel and injected subcutaneously into male nude mice to successfully create a vascular model resembling a hemangioma. A month later, a decrease in blood vessels and the emergence of adipocytes were observed, representing the regression process. The results indicated that HemEC may exhibit stem cell-like phenotypic and functional characteristics, as well as clonality and multipotency.

### HemECs combined with HemPericytes model

During the proliferation phase of IHs, pericytes surrounding newly formed blood vessels typically express α-smooth muscle actin (α-SMA), neural/glial antigen 2 (NG2), platelet-derived growth factor receptor (PDGFR), calponin, and smooth muscle myosin heavy chain transmembrane receptor protein 3 (NOTCH3), which are typically associated with smooth muscle cells [60, 61]. Thus, the pericytes that surround blood vessels in IH are typical of HemPericytes and smooth muscle cells. They have also been shown to have mesenchymal stem cell characteristics [62]. Boscolo et al. [63] isolated HemPericytes from different patients’ IH samples during the proliferation and involution phases. HemPericytes immunohistochemistry revealed that NG2, PDGFR, α-SMA, NOTCH3, and other markers were positive in vitro. After 7 days, new blood vessels could be seen when HemPericytes were combined with endothelial cells and implanted into naked mice. IH HemPericytes proliferated faster and expressed more VEGF-A than normal human HemPericytes isolated from the retina or placenta, but the level of angiopoietin 1 (ANGPT1) was significantly lower. HemPericytes from vascular tumors inhibited the proliferation and migration of normal human endothelial cells in coculture. As a result, the increase in VEGF-A, decrease in ANGPT1, increase in in vivo vascular formation, and increased ability to inhibit endothelial cell proliferation and migration all point to HemPericytes promoting angiogenesis in IH. We successfully established an IH model in our recent study [64] by injecting HemECs and HemPericytes into the subcutaneous tissue on the back of BALB/C-nu male mice. Molecular biology and cell biology experiments revealed that knocking down 6-phosphofructo-2-kinase/fructose-2,6-bisphosphatase 3 (PFKFB3) could inhibit IH vascularization and migration by affecting glycolytic metabolism and inducing cell apoptosis via apoptotic pathways, implying that targeting PFKFB3 could be a new treatment strategy for IH. Recent research has also shown that, in addition to the paracrine secretion of VEGF-A, HemPericytes can stimulate autocrine VEGF-A expression by tumor endothelial cells, which may inhibit endothelial cell apoptosis [65]. HemSCs and HemPericytes, surprisingly, secrete high levels of VEGF-A, promoting angiogenesis [66].

## Transviral and genetic models

### Polyoma virus transfection model

**Fig. 3:**
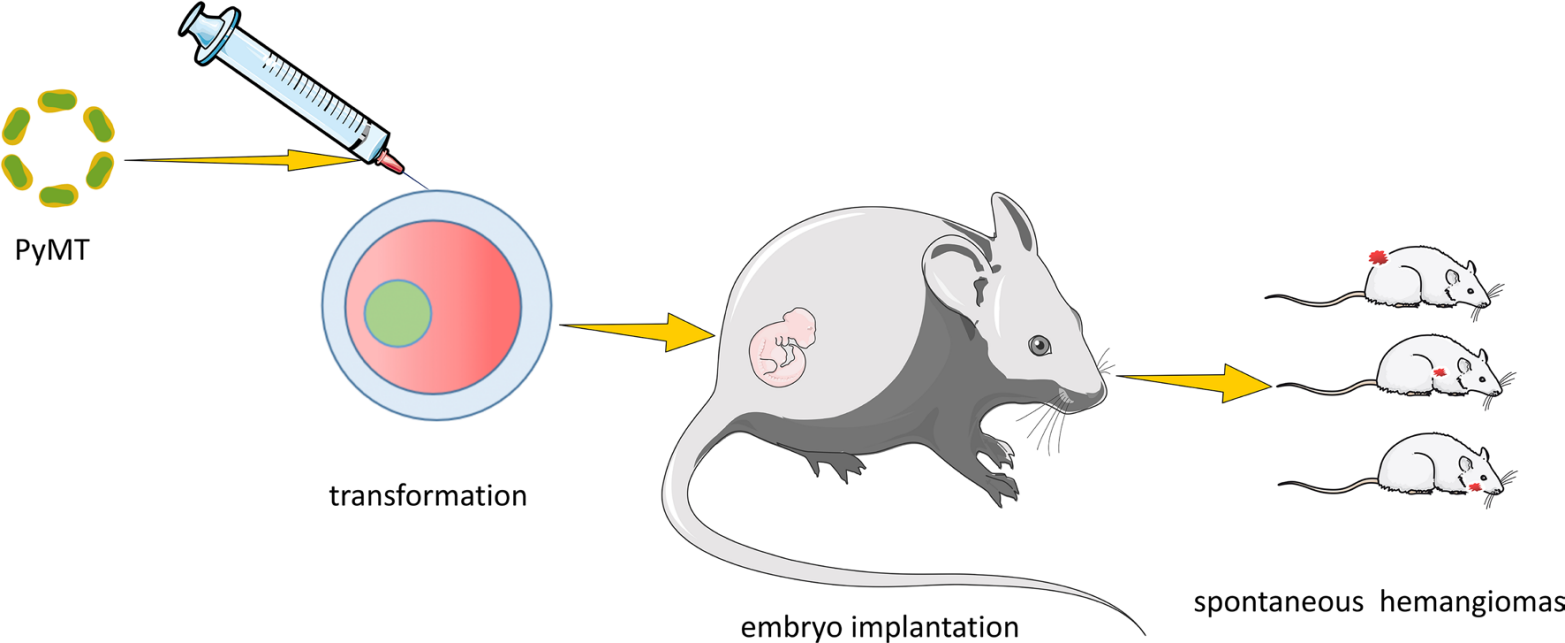
Transviral model. The animal model of IH was established by microinjection of PyMT transgenic DNA. The constructed transgenic PyMT was injected into fertilized mouse embryos and then transferred into the uterus of pseudopregnant mice. After birth, it was found that the mouse IH model of transgenic PyMT DNA was successfully constructed, and IH was mainly distributed in the skin surface, tongue, ear mucosa and stomach mucosa. The PyMT gene was also expressed in transgenic mouse DNA.

In 1953, a small deoxyribonucleic acid (DNA) virus called murine polyomavirus (MPyV) was found. The virus is made up of the middle T oncogene (PyMT), which is a transforming endothelial oncogene that can recruit and cause endothelial progenitor cells to change and become immortal [67]. The PyMT gene produces a protein that is similar to a human protein and can act as a tyrosine kinase binding site and an active cell membrane receptor. This turns on intracellular and extracellular signaling pathways, helps hemangiomas grow in mice, and mimics the way human infantile hemangiomas grow [68]. Many studies have shown that PyMT is a key player in the growth of tumors and can cause cell death in vitro, which can lead to benign and malignant tumors in organs such as the skin, salivary gland, and mammary gland of mice [69, 70]. Xu et al. [71] made an IH animal model by microinjecting the PyMT transgenic DNA of polyomavirus into fertilized embryos, transferring them to mice that looked like they were pregnant, and observing the tumors in the newborn mice’s phenotypes and histological shapes (Fig. 3). The results showed that the PyMT transgenic DNA IH model was built correctly and that the PyMT gene was expressed in the transgenic mouse DNA. Xu then made a transgenic mouse with the PyMT gene driven by the SV40 promoter. Surviving fertilized eggs microinjected with the PyMT gene showed an IH phenotype that had the PyMT gene and expressed it. Tumors in IH phenotype transgenic mice had a sponge-like hemangioma structure, with abnormal vascular growth on the surface of the skin, tongue, ear mucosa, and stomach mucosa. Ki-67 staining with immunohistochemistry showed that the tumor was IH and not angiosarcoma. All of the PyMT transgenic mice lived for only 4 weeks. PyMT driven by the SV40 promoter is a better model because it can cause IH. The problem is that it kills mice after they give birth. Bussolino [72] injected the PyMT gene into mice and established a stable tumor endothelial cell line from blood vessel endothelial cells. Liekens [73] created an IH model by injecting MPyV intraperitoneally into 4-day-old mice. They discovered that the skin, muscles, and cranial brain were all involved and that the number and size of IHs increased exponentially and were associated with bleeding and anemia, which histologically matched human cavernous IHs. Using SCL-TVA mice and recombinant RCAS/PyMT virus, Sausville [74] successfully delivered the PyMT virus to endothelial progenitor cells in blood vessels. They discovered that infected mice died quickly from hemorrhagic disease caused by the formation of IH, and this could be because endothelial cells transformed into IH stem cells after being infected with PyMT. Thus far, it is unclear why PyMT promotes IH. Certain cytokines, such as tyrosine kinase, Scr family kinase, and nitric oxide (NO), have been found to have signal transduction functions in some studies [75]. The benefit of this model is that it directly forms IH and is primarily distributed in areas such as the skin and mucosa, and tumors can also be formed in immunocompetent animals, allowing researchers to study the immune system’s effects on tumor cells and the microenvironment. The disadvantage is that the histological characteristics of IH caused by PyMT are usually malignant and resemble vascular tumors or vascular sarcomas.

### VEGF model and basic fibroblast growth factor (BFGF) model

**Fig. 4:**
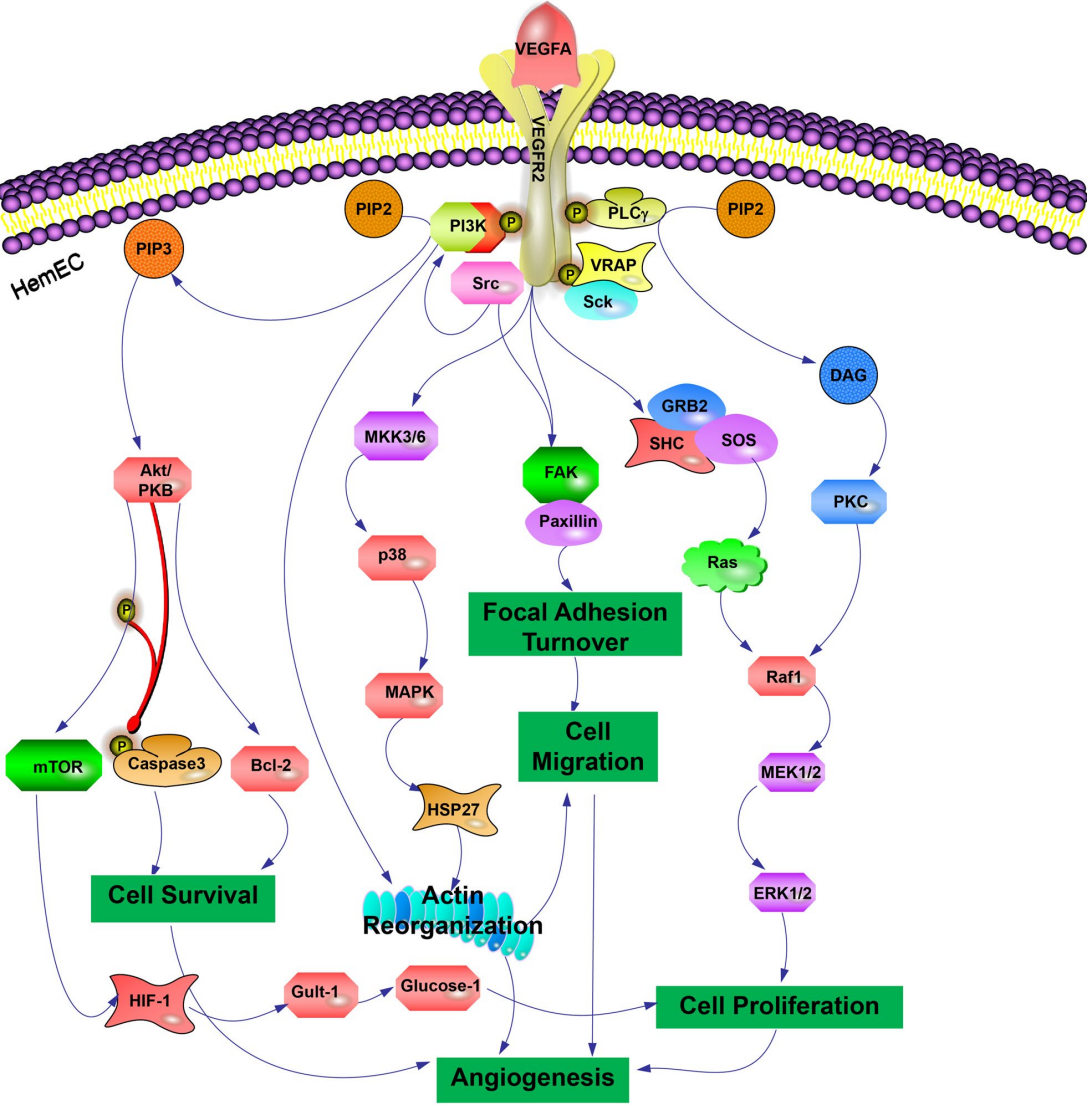
The VEGF-A/VEGFR-2 signaling pathway promotes angiogenesis in IH. VEGF-A promotes angiogenesis by activating the VEGFR-2 receptor in HemECs by triggering multiple downstream signaling pathways, including microvascular permeability, HemEC proliferation, migration, and survival. Activation of VEGFR-2 in HemECs triggers multiple downstream signals that promote angiogenesis. These pathways are as follows: (1) VEGFR-2 activates mitogen-activated protein/Ras/Raf1/ERK/MEK signaling pathways, which promote HemEC proliferation and thus promote angiogenesis. (2) The activation of the PI3K/serine-threonine protein kinase/Akt signaling pathway by VEGFR-2 promotes cell survival. VEGFR-2 also activates the Akt/mTOR/HIF-1/Gult-1/Glucose-1 signaling pathway and increases glycolysis to promote HemEC proliferation and thus promote angiogenesis. PI3K/Akt activation can promote the expression of Bcl-2 and play an anti-apoptotic role to promote cell survival. (3) VEGFR-2 can directly promote angiogenesis by activating the MKK/p38/MAPK/HSP27 signaling pathway and affecting intracellular actin recombination. (4) The VEGFR-2 receptor directly activates FAK/Paxillin to promote cell migration and thus generate neovascularization. These signaling pathways play different cellular biological functions but also have synergistic effects on each other to promote the generation of neovascularization. Mechanistic target of rapamycin (mTOR), hypoxia-inducible factor-1 (HIF-1), mitogen-activated protein kinase kinase (MKK), heat shock protein 27 (HSP27)

**Fig. 5:**
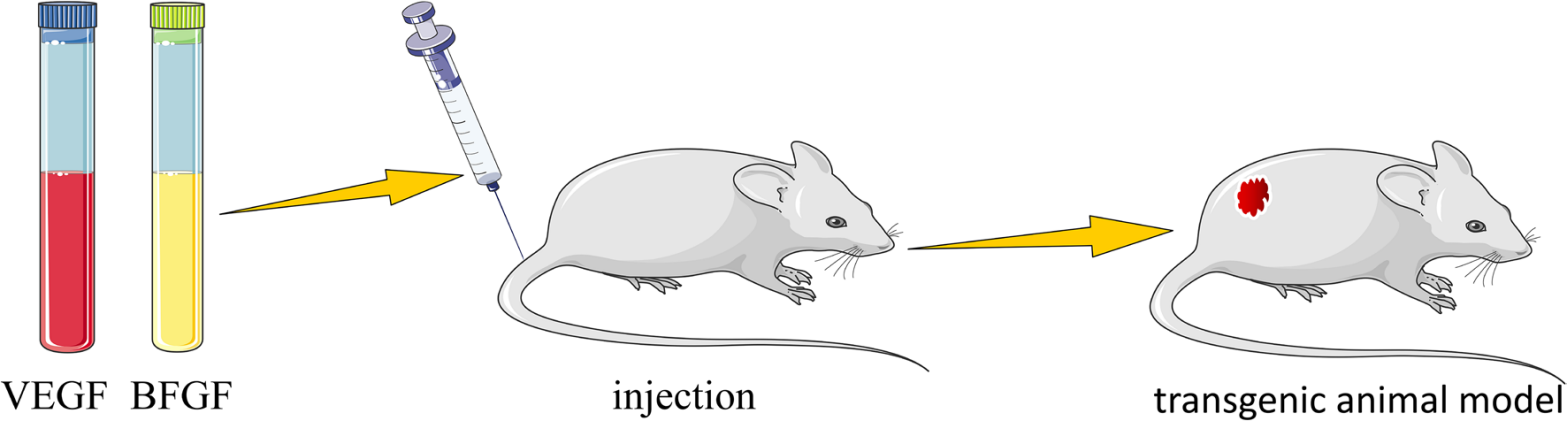
Transgenic model. When injected into mice, VEGF and BFGF activate HemECs and then form hemangiomatous lesions locally

The VEGF family includes VEGF-A, VEGF-B, VEGF-C, VEGF-D, and placental growth factor (PIGF). These growth factors are essential for embryonic development and angiogenesis [76, 77]. According to research, VEGF expression in IH is proportional to its proliferation (Fig. 4) [78]. On the other hand, VEGF expression rapidly decreases during regression, and angiogenesis is significantly suppressed [79, 80]. In a mouse study, Lee et al. [81] discovered that VEGF overexpression in local gene therapy for ischemic disease can result in the formation of vascular tumor-like lesions (Fig. 5). Tajima et al. [82] established a rabbit model using transgenic technology to study the relationship between VEGF165 and atherosclerosis under the control of the human antitrypsin promoter. The transgenic rabbits exhibited hepatosplenomegaly, vascular network formation, and diffuse IH, according to the findings. Furthermore, the transgenic rabbits developed symptoms resembling Kasabach-Merritt syndrome (KMP), including hemolytic anemia, thrombocytopenia, and splenomegaly. Clinical studies have also revealed that high VEGF expression is linked to a variety of pathophysiological conditions, including IH, because it can activate endothelial cells and promote neovascularization [83]. One of the most important growth factors in angiogenesis is BFGF. By binding to fibroblast growth factor receptor 1 (FGFR1) on the surface of target cells, BFGF induces phosphorylation, which plays a role in many signaling pathways, including cell proliferation, differentiation, and angiogenesis [25, 84]. Gualandris [85] transfected murine aortic endothelial cells with a retroviral expression vector containing human BFGF cDNA and injected the cells into naked mice, resulting in Kaposi’s sarcoma-like angioproliferative lesions. Angiogenesis was induced in avascular rabbit corneas by injection. Injection into chick embryos increased vascular density and resulted in chorioallantoic membrane vascular tumors. Overexpression of BFGF in endothelial cells resulted in a pro-angiogenic phenotype and recruitment of dormant endothelial cells, resulting in vascular proliferative lesions. Choroszczak et al. [86] investigated changes in VEGF and BFGF serum concentrations in 51 IH patients during propranolol treatment and discovered that serum concentrations of both factors were significantly reduced, possibly due to their inhibitory effects on angiogenesis, induction of endothelial cell apoptosis, and vasoconstriction. This indirectly indicates the model’s viability. These findings suggest that this model could be useful for studying the pathogenesis and complications of IH, as well as inhibiting angiogenesis. The disadvantage is that the high technical requirements for building this model result in unstable results.

## Tissue block transplantation model

**Fig. 6:**
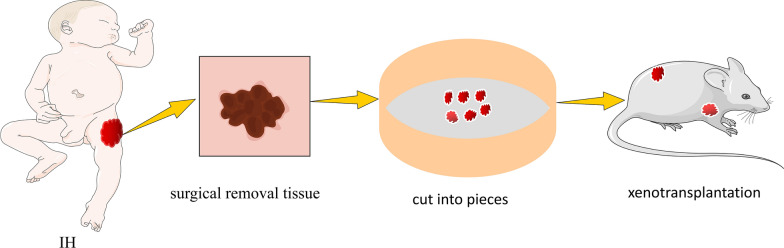
Tissue block transplantation model. Human IH tissue was xenografted subcutaneously into the back of nude mice without the thymus. Due to the loss of cellular immune function caused by the lack of T lymphocytes in nude mice, the transplanted human tissue could survive and proliferate in nude mice, thus building a xenograft tissue transplantation model

Xenograft transplantation is the transplantation of human normal or tumor tissue into immunodeficient nude mice. The absence of T lymphocytes in nude mice results in a loss of cell-mediated immunity, which permits human tissue to survive in the host and establish new circulation within the host, making it a suitable vehicle for constructing various disease or tumor models [87]. First utilized by Tang et al. [88] to create an IH animal model. They obtained IH tissue samples that were proliferating from a 2-month-old boy, cut them into small pieces, and transplanted them subcutaneously into immunodeficient nude mice. After the initial ischemic phase, the majority of transplants exhibited rapid growth, followed by gradual regression and replacement by fibrofatty tissue. By injecting nude mice with a large dose of estrogen every week, they obtained a tumor that was more ideal in terms of tumor volume, growth characteristics, and duration (Fig. 6). This result indicated that estrogen can promote the development of IH. Immunofluorescence staining revealed that the majority of endothelial cells in nude mice were derived from human IH tissue, whereas mouse endothelial cells appeared earlier but primarily around the transplanted tissue. This phenomenon suggests that the transplant’s blood supply system is dependent on the neovascularization of human endothelial cells in IH tissue. Moreover, xenografted vascular tumors may maintain a degree of independence from the host and may not be replaced by host cells for a relatively lengthy period of time. This model has the ability to simulate all the biological characteristics of human IH, retains the histological, molecular, and genetic characteristics of human IH, and can be used to determine the tumorigenicity, invasiveness, and drug sensitivity of tumor cells. It must be performed on immunodeficient animals, it cannot reflect the dynamic process of tumor immune surveillance, it has high requirements for tissue blocks, necessitating immediate operation after specimen collection, and large quantities of models cannot be obtained simultaneously. Some transplanted tissues regress rapidly in nude mice without obvious proliferation, and the number and density of newly formed blood vessels cannot reach the optimal level. This may be because IH is a benign tumor, its invasiveness decreases after transplantation into nude mice, and its growth rate is slow; another possibility is that IH tissue blocks die quickly in nude mice due to a lack of nutritional supply and cytokine stimulation.

## Three-dimensional (3D) model of cell culture in vitro

The tumor microenvironment, which is composed of HemPericytes and extracellular matrix (ECM) in addition to tumor cells, is present in all tumors. Cell migration, proliferation, and differentiation are all significantly influenced by the ECM [89]. Building an in vitro model that closely resembles the in vivo tumor environment is crucial because the tumor microenvironment is incredibly complex [90]. Through extensive research, researchers gradually came to understand the significance of the microenvironment in influencing the morphology, phenotype, structure, and function of tumor cells, starting with Paget’s “seed and soil” theory [91]. IH cell morphology and structure, however, change in conventional two-dimensional cell culture and cannot faithfully mimic the in vivo environment. Large-scale cell-level and molecular-level studies are challenging to carry out because, despite the limitations of experimental animals themselves, animal models can accurately reflect the in vivo growth and proliferation of IH cells but cannot accurately reflect the characteristics of the human IH phenotype and drug treatment response [92]. Therefore, we developed a novel three-dimensional cell in vitro culture system to more accurately mimic the growth process of IH cells in vivo. The needs of interactions between cells and between cells and the matrix can be met by three-dimensional cell culture technology, which can also give IH cells an environment for growth in vitro that is similar to that found in vivo [93]. To improve the structure and function of mesenchymal scaffolds in various tissue applications, to repair the vascular system after injury, to improve the recovery of parenchymal tissue after injury and to produce and deliver soluble proteins, the implantation of porous scaffolds into various cell models has gradually been used in tissue regeneration medicine in recent years [94, 95]. In addition, implantation of cells into a three-dimensional scaffold has been used to improve cell survival, including processes such as angiogenesis, cell migration, invasion, differentiation, and tumor formation, by creating a microenvironment similar to that of the human body [96, 97]. Synthetic, natural, and bioderived hydrogels can be utilized for 3D encapsulated tumor cell culture, reconstructing the ECM microenvironment in vitro. Hydrogels have strong water-retention characteristics and a porous microstructure, allowing for the efficient transport of nutrients and metabolic waste during cell culture [98]. The network structure of hydrogels provides a biomimetic matrix for cells, thereby inducing cell-matrix interactions. The porous structure is advantageous for cell migration and intercellular signaling, inducing the formation of cell spheroids, making it an ideal model for 3D tissue culture [99]. Collagen, matrix gel, and alginate are the most frequently used biological source hydrogels as extracellular matrix proteins. These biologically derived hydrogels are more compatible with 3D cell culture [100].

### Fibrinogen gel model

The creation of a three-dimensional IH in vitro model using fibrin gel has become more popular as IH research develops. An innovative in vitro culture system that embedded a small IH biopsy fragment in a fibrin gel well and cultured it in serum-free medium was first described by Tan et al. in 2000 [101]. Consequently, a complex microvascular network grew from the tissue fragment and underwent the three phases of IH development: a microvascular platform phase from days 1 to 4, a proliferative phase from days 5 to 7, and a regressive phase from days 7 to 12. This model offers a novel method for clarifying the biological behavior of IHs. The model avoids the disadvantages of animal models, and its cellular and molecular mechanisms are more similar to those of human IH. In addition, new blood vessels can develop in the absence of exogenous serum, and experimental research can involve the addition of drugs such as growth factors and antibodies.

### Hydrogel endothelial cell implantation model (Fig. 7A)

**Fig. 7:**
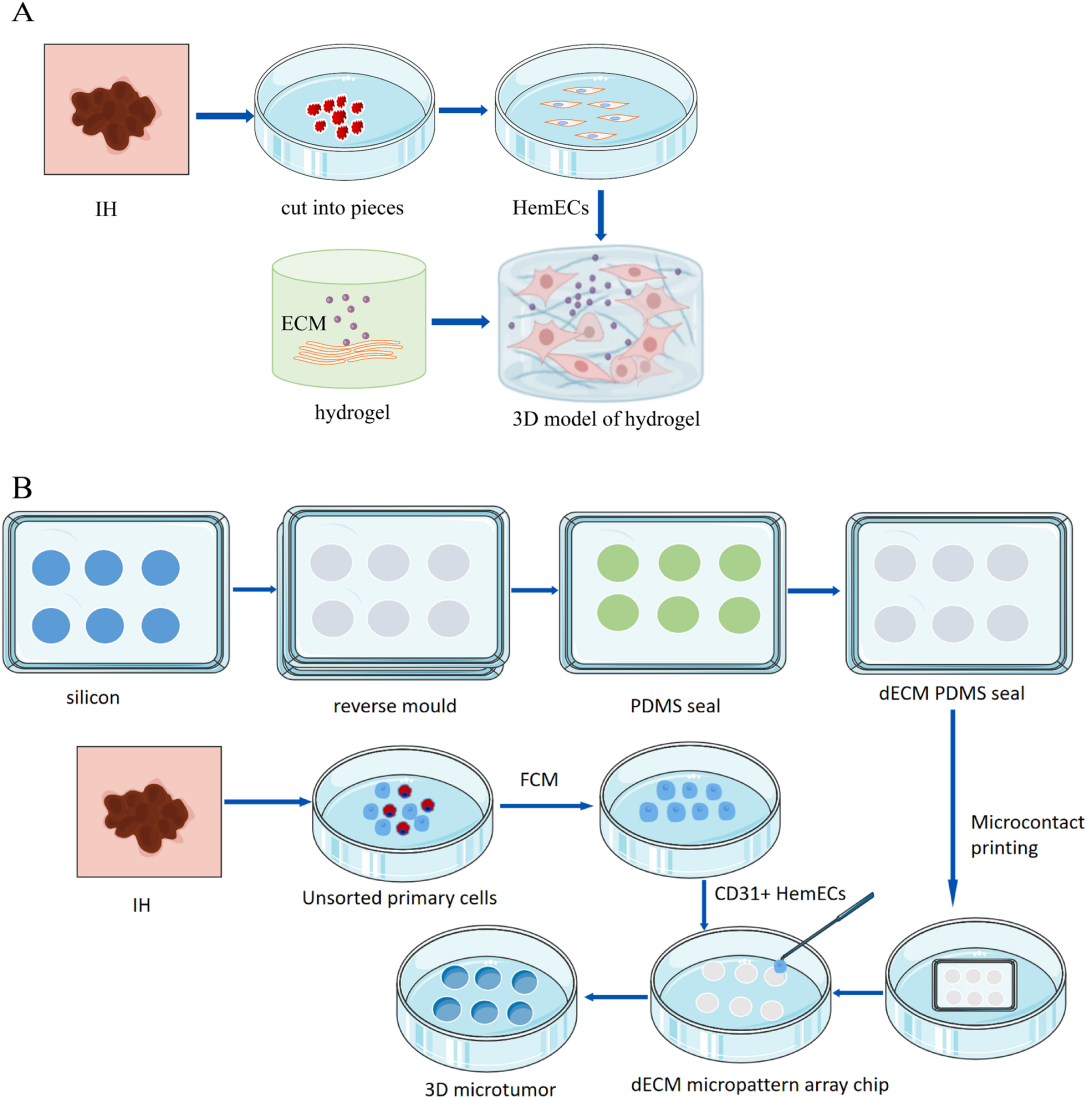
**A** 3D model of the hydrogel. A. After the human IH tissue was cut into small pieces, HemECs were isolated by flow cytometry, and a three-dimensional hydrogel scaffold with ECM was prepared at the same time. Finally, HemECs were implanted into the three-dimensional scaffold for culture, and the hydrogel 3D IH model was constructed. **B** 3D microtumor model. Primary IH endothelial cells were isolated and cultured, and CD31+ HemECs were obtained by flow cytometry. At the same time, DAM was obtained by using tissue engineering to DAM, and DAM solution was prepared to provide specific ECM for cell culture. Then, PDMS chips with different diameters are obtained by etching characteristic patterns on silicon wafers by laser. Finally, CD31+ HemECs were plated on micropattern array petri dishes to construct 3D microtumor models

Tsuneki et al. reported the development of an IH model using endothelial cell implantation in a hydrogel in 2015 [102]. The researchers cultured vascular endothelial cells in a three-dimensional hydrogel scaffold before implanting them subcutaneously in mice to form a microvascular network that eventually developed into a structure similar to IH. These implants formed a block-like structure after 4 weeks, similar to an expanded vascular plexus. This model is one of the most promising models for research in the fields of angiogenesis, 3D cell culture, vascular growth factors, and cell-to-cell communication [103, 104]. It resembles the progression of IH observed in infants and young children, from the proliferative to the regressive stage. The hydrogel 3D encapsulation culture of tumor cell spheres has the advantage of providing cells with a 3D microenvironment, whereas the biologically derived hydrogel is expected to reconstruct tumor cells’ ecological niche in vitro [105]. This encapsulation culture, however, has drawbacks such as difficult observation and analysis, as well as inconsistent sizes and shapes of tumor cell spheres. Because the process of angiogenesis and regression is brief and the budding time is difficult to control, synchronized intervention experiments are difficult to carry out. Furthermore, the blood vessel wall of this model is made up of a single layer of flattened endothelial cells, and the surrounding tissue is mostly empty and vacuolar, which differs significantly from the proliferative stage of human IH tissue, making it unsuitable for dynamic observation studies.

### 3D microtumor model

The simplest 3D in vitro model is the tumor spheroid, which is composed of multicellular structures [106]. Tumor spheroid models for various research purposes have been developed, including nonvascularized tumor research models, tumor drug resistance research models under hypoxic conditions, and research models that combine with microfluidics to study the shear force of blood and tissue fluid for tumor drug resistance [107]. The tumor spheroid model mimics many aspects of in vivo tumors, such as cell interactions, hypoxia, central necrosis, and drug resistance, and has been widely used in drug development research [108]. Recent research has shown that 3D tumor spheroids improve cell function, tissue morphology, vitality, genotype stability, and drug metabolism. Their cell aggregates are coated with natural ECM and are more similar to in vivo tumors in a 3D environment [109, 110], potentially filling the gap between two-dimensional (2D) culture models, animal models, and patient-derived xenograft (PDX) models [111]. As a result, the development of 3D tumor spheroid models with controllable size and ordered layout is advantageous for IH pathogenesis research and high-throughput drug screening.

Recently, some researchers developed microarray patterns in culture dishes in which cells are restricted to adhere to micropatterns and can form 3D structures resembling spheres via cell proliferation and intercellular adhesion. The structures’ size and shape can be adjusted [112]. Previously, we isolated and cultured primary IH endothelial cells and obtained CD31+ HemECs using flow cytometry [113]. Based on this, our team first used tissue engineering technology to decellularize pig main arteries, resulting in a decellularized artery matrix (DAM) solution that provided specific extracellular matrix for cell culture. Then, using a laser, we etched feature patterns on silicon wafers to create polydimethylsiloxane (PDMS) chips of various diameters. Finally, we seeded CD31+ HemECs on a micropattern array culture dish to create a 3D microtumor model (Fig. 7B) [114]. We observed the formation of CD31+ HemEC spheres in the micropattern array using this model and discovered that the appropriate diameter for cell morphology, vitality, proliferation, and phenotype expression was 100–150 μm. In the future, we will use 100 μm micropattern arrays for mechanistic research and drug screening. The structure of the micropattern array culture dish is relatively definite in this model, and the structure can be directly linked to the function. This system’s cellular morphology and signal transduction processes are closer to the physiological state and can be monitored in real time. Furthermore, the interaction between cells in this system, as well as the interaction between cells and the extracellular matrix, can more accurately reflect changes in the tumor microenvironment and cell morphology, and good cell morphology and the surrounding microenvironment are important for cell behavior and gene expression [115]. Meanwhile, ECM can mimic the loose or dense connective tissue that surrounds cells, allowing researchers to investigate tumor cell proliferation, metastasis, invasion, and other behaviors [116]. Tumor cell spheres exhibit drug sensitivity similar to in vivo tumors. Using tumor cell spheres for drug screening prior to conducting animal experiments can significantly improve animal experiment success rates [117]. The disadvantage of this model is that it primarily simulates spherical tumors, whereas tumors in vivo can take on a variety of shapes. Furthermore, the method’s preparation is complex, and it can usually only simulate a static environment, whereas the microenvironment in vivo is constantly changing.

There are some differences between the Matrigel and 3D models. Matrigel, a material secreted by Engelbreth-Holm-Swarm mouse sarcoma cells, is rich in ECM protein [118], as well as a variety of growth factors and matrix metalloproteinases [119]. It has been widely used in the colon, stomach and liver [120]. Matrigel has wide application range and low cost. In addition, Matrigel has the following disadvantages: (1) the differences between batches of Matrigel are large and difficult to control, and the nutrients and protein components contained in Matrigel are greatly affected by batches [121–123]; (2) due to its temperature sensitive characteristics, Matrigel has strict temperature requirements for storage and operation links, etc.; (3) Matrigel has certain toxicity and is limited in the application of drug screening; (4) poor mechanical property control [124]; (5) the fact that Matrigel is derived from mouse cells prevents its clinical use in humans due to its potential immunogenicity [125]. However, the 3D model currently uses animal-derived aortic acellular matrix, which has a three-dimensional frame structure and intercellular attachment effect, providing more growth space for cell migration and proliferation. In addition to retaining various active components and growth factors in the ECM, 3D model can also promote cell repair and cell function recovery. Compared with Matrigel, acellular matrix has more advantages in cytocompatibility, biocompatibility, immunogenicity and mechanical properties [126, 127]. In addition, the 3D microtumor model also has the following advantages: (1) it can retain the collagen structure in the extracellular matrix, has low immunogenicity, good biocompatibility and mechanical properties, and can promote cell adhesion growth and tissue regeneration; (2) no toxic products, no immunogenicity or low immunogenicity; (3) the complex three-dimensional porous microstructure of tissues is preserved to ensure the effective transportation of nutrients and metabolic wastes during cell culture [128]; and (4) excellent biocompatibility and good biomechanical properties.

## Conclusions and future directions

Various types of IH models are continuously evolving and improving in tandem with the advancement of medical technology. Multiple factors regulate the occurrence and progression of IH, which is a complex pathological process. Establishing an ideal animal model of IH provides a solid experimental foundation for studying the pathogenesis and mechanisms of IH, which is of critical clinical importance for the discovery of new drugs, treatments, or approaches. Each type of IH model has its own advantages and disadvantages, so the optimal choice must be based on the research’s subject matter and objectives. The cell suspension injection method is relatively simple and cost-effective, can obtain a large number of cells through in vitro cloning, and the tumor maintenance time is relatively long, allowing for some long-term drug intervention studies. However, this procedure implants a single cell into the mouse’s body. Although HemSCs are capable of differentiating in multiple directions and can be induced to differentiate in a particular direction by differentiation inducers, the direction and degree of differentiation in the mouse body cannot be fully controlled. The viral gene model is induced to produce at the gene level, and the cell components and tissue structure of the tumor are formed naturally. However, the source of the tissue is mice, the distinction between the formed tumor and human hemangioma is unclear, and the formed tumor is predominantly malignant or prone to bleeding, with a low survival rate in mice. The tissue block transplantation method obtains the tumor directly from human tissue, thereby preserving the histological structure and biological characteristics of the IH to the greatest extent possible. However, obtaining samples is challenging, and adding intervention factors directly affects IH tissue block, whereas body circulation metabolism and the tumor microenvironment have no effect on local tumors. The greatest advantage of our team’s 3D tumor cell sphere model is the ability to control the tumor’s size, dimensions, and layout arrangement. In addition, the ECM creates conditions for cell proliferation, migration, and differentiation. However, the main limitation of this model is that it mainly simulates spherical tumors, while the morphology of tumors in the body is diverse. In addition, the preparation process of this method is relatively complex and can only simulate a static environment, while the microenvironment in the body is constantly changing. Therefore, these models cannot fully simulate the natural process of human IH growth and regression and cannot fully demonstrate the pathological characteristics of human tissues. As human IH does not occur in other species, it increases the difficulty of establishing animal models. The ideal IH model must first consider the two key periods of IH proliferation and regression and accurately reflect the biological behavior and molecular basis of IH. Second, it should also include the immune microenvironment and ECM involved in IH growth. Finally, the construction process should be simple, easy to operate, and suitable for large-scale production. However, with the development of biomaterials and 3D imaging technology, a sustainable and integrated culture system of tumor cell spheres can be continuously developed. At the same time, the development of standardized procedures for culturing tumor cell spheres can improve the authenticity, stability, and credibility of experimental results. Tumor cell spheres not only reproduce some features of in vivo tumor tissues but also have low batch-to-batch variability and can achieve high-throughput automated analysis and compatibility with various 3D imaging technologies. Therefore, they have tremendous potential in preclinical drug screening.

**Fig. 8:**
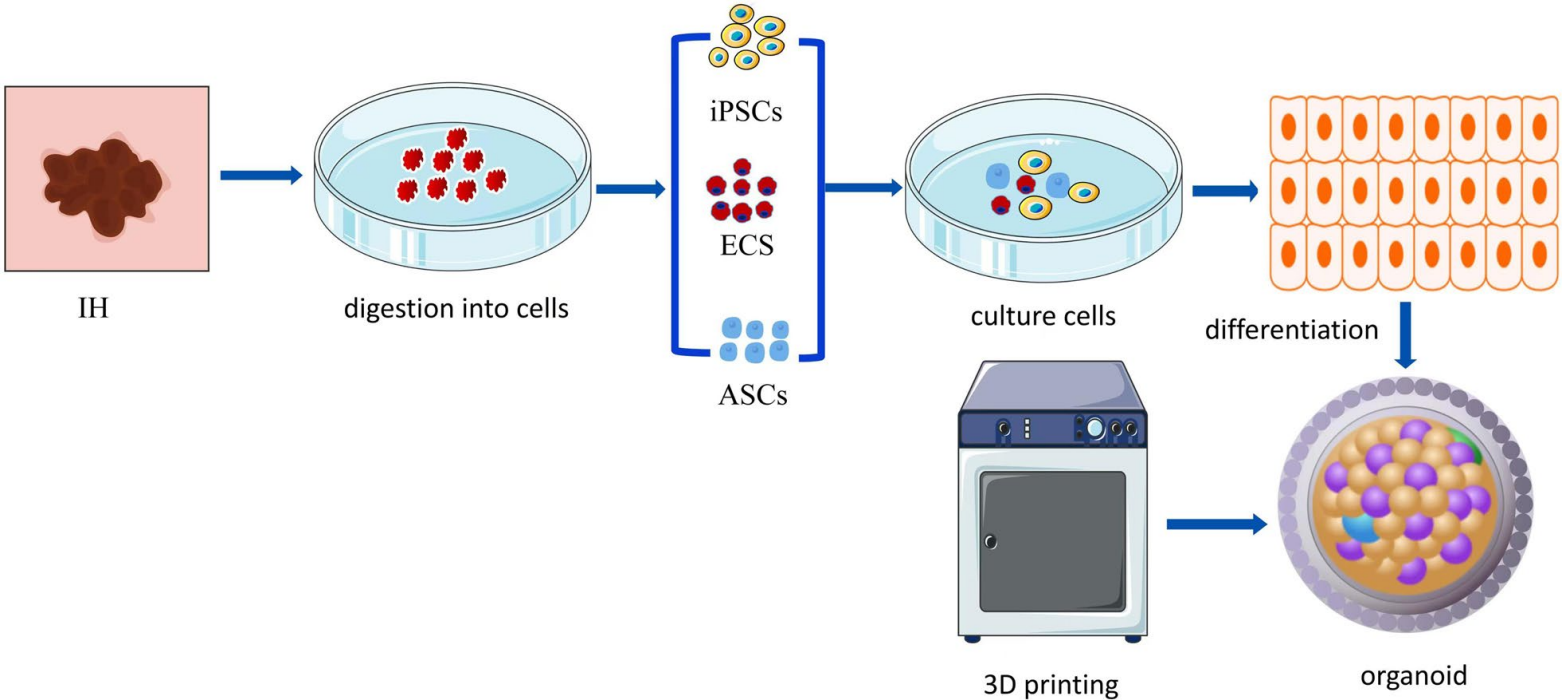
Organoid model. Human IH tissue was cut into small pieces, and ESCs, iPSCs and ASCs were isolated by flow cytometry and differentiated into functional cell types of micro cell clusters through cell culture technology, simulating the structure and function of human skin IH. At the same time, 3D bioprinting technology can produce a variety of different specifications of vascular systems to promote nutrient tissue absorption and control the size of the model. neonatal or adult stem cells (ASCs).

Organoid models have appeared as a result of the advancement of 3D cell culture techniques, research into ECM, and stem cell niches. Cystic teratomas were used by Smith et al. to define “organoids” for the first time in 1946 [129]. Today, however, the term “organoids” refers to microcell clusters that develop in vitro in a 3D environment, form tissues, and differentiate into distinct cell types that mimic the form and function of organs in the human body [130]. Organoids can be produced using induced pluripotent stem cells (iPSCs) or neonatal, adult, or embryonic stem cells (ESCs) [131]. Organoids closely resemble the 3D structure, cell type composition, and function of real organs when used as a 3D in vitro culture model (Fig. 8). Organoids maintain both the benefits of streamlined and accessible cell culture models while retaining the heterogeneity of distinct tumors. In basic research, disease modelling, drug screening, and regenerative medicine, organoids have proven to be extremely valuable [132]. Organoid systems prepared using different types of stem cells and pluripotent cells from animals and humans have been applied to a variety of tissues, including the thyroid, pancreas, small intestine, lungs, liver, heart, and brain. This has led to new ideas for the precise treatment of tumors and the personalized care of patients [133]. There are currently no reports of organoids in IH, but Wimmer et al. [134] induced human embryonic stem cells (hPSCs) into mesodermal cells in the cardiovascular system, which could be further induced to form vascular organoids. Endothelial cells and hempericytes assemble into cell complexes to form these vascular organoids. In the mature vascular organoids, a capillary network enveloped by a basal lamina was established. Vascular organoids can form a stable vascular tree consisting of arteries, arterioles, and venules following transplantation. With the rapid advancement of medicine, organoids are frequently derived from patients due to their multiple benefits [135]. Organoids derived from patients can retain the drug resistance and gene mutations found in the original tissue [136]. These can replace tumor cell lines, animal models, and tumor xenografts [137]. They can also serve as a biological sample library for drug development [116]. Of course, there are still some limitations to this technology, such as the limited maturity and cell diversity of in vitro organoids when replacing human organs, the unsuitability of this technology for organ transplantation and large-scale production, and the inability to fully replicate in vivo tissue interactions due to defects in the vascular, nervous, and immune systems. In addition, issues such as differentiation and variation during passage remain. Coculturing with HUVECs derived from iPSCs is the current solution for this issue; however, HUVECs may cause immune rejection [138]. Furthermore, 3D bioprinting technology has the potential to improve organoid culture technology. 3D bioprinting, as a promising new technology, can print cells or biocompatible components into complex tissues by using appropriate cell frameworks and topological structures, resulting in greater cell specificity and good separation characteristics for the designed structures [139]. This technology is better suited to support the growth and maturation of different cell types while preserving their overall cell diversity [140]. Furthermore, 3D bioprinting can create a variety of vascular systems to improve nutrient absorption and size control [141]. Organoids can become in vitro functional organs with different cell types, blood vessels, and nervous and immune systems under certain conditions. 3D bioprinting is currently possible using three techniques: biomimetics, self-assembly and microtissue building blocks [142]. 3D bioprinting is now widely used in a variety of functional tissues, such as skin, bone, respiratory tissue, heart tissue, cartilage, and vascular tissue [143]. Furthermore, because these models are superior to animal models, tissues generated by this technology can serve as ideal models for drug discovery, analysis, and screening [144]. As a result of our deep understanding of stem cell biology and developmental biology, we can anticipate new organoid technologies that can comprehensively construct models of the structure and function of human organs, thereby promoting disease basis and clinical research.

## Data Availability

Not applicable.

## Abbreviations

3D: Three-dimensional
IH: Infantile hemangioma
RAS: Renin-angiotensin system
HemSCs: Hemangioma stem cells
HemECs: Hemangioma endothelial cells
HemPericytes: Hemangioma-derived pericytes
GLUT-1: Glucose transporter-1
GFP: Green fluorescence protein
SALL4: Spalt-like transcription factor 4
VEGF: Vascular endothelial growth factor
β-AR: β-Adrenergic receptor
RTK: Receptor tyrosine kinase
cAMP: Cyclic AMP
MAPK: Mitogen activated protein kinases
ERK: Extracellular signal regulated kinase
MMS: Monocyte-macrophage cell
PKB: Protein kinase B
ERK1/2: Extracellular signal-regulated kinase 1/2
HUVECs: Human umbilical vein endothelial cells
PKM2: Kinase M2 isoform
α-SMA: α-Smooth muscle actin
NG2: Neural/glial antigen 2
PDGFR: Platelet-derived growth factor receptor
NOTCH3: Transmembrane receptor proteins 3
ANGPT1: Angiopoietin1
PFKFB3: 6-Phosphofructo-2-kinase/fructose-2,6-bisphosphatase 3
DNA: Deoxyribonucleic acid
MPyV: Murine polyomavirus
PyMT: Middle T oncogene
NO: Nitric oxide
BFGF: Basic fibroblast growth factor
PIGF: Placental growth factor
KMP: Kasabach-Merritt syndrome
FGFR1: Fibroblast growth factor receptor 1
ECM: Extracellular matrix
2D: Two-dimensional
PDX: Patient-derived xenograft
DAM: Decellularized artery matrix
PDMS: Polydimethylsiloxane
iPSCs: Induced pluripotent stem cells
hESCs: Human embryonic stem cells
